# The complete chloroplast genome sequence of *Campanula zangezura* (Campanulaceae)

**DOI:** 10.1080/23802359.2019.1704658

**Published:** 2020-01-10

**Authors:** Kyung-Ah Kim, Ki-Oug Yoo, Kyeong-Sik Cheon

**Affiliations:** a Department of Biological Sciences, Kangwon National University, Chuncheon, South Korea;; b Environmental Research Institute, Kangwon National University, Chuncheon, South Korea;; c Department of Biological Science, Sangji University, Wonju, South Korea

**Keywords:** Campanulaceae, *Campanula*, *Symphyandra*, chloroplast genome, phylogeny

## Abstract

The complete chloroplast genome sequence of *Campanula zangezura* was determined by Illumina pair-end sequencing. The complete cp genome was 166,331 bp in length, containing a large single-copy (LSC) region of 100,034 bp and a small single-copy (SSC) region of 7781 bp, which were separated by a pair of 29,258 bp inverted repeats (IRs). A total of 113 unique genes were annotated, including 79 protein-coding genes, 30 tRNA genes, and 4 rRNA genes. Among these genes, 17 genes contained one or two introns. The ML tree based on 74 protein-coding genes showed that *C. zangezura* formed a sister to the *Campanula punctata* and *Campanula takesimana* clade.

The sect. *Symphyandra* was considered to be an independent genus due to the character of the connate anther. At present, however, it is commonly considered to belong to the genus *Campanula* due to the formed polyphyly in the Campanula species clade (Eddie et al. 2003; Haberle et al. 2008; Raab-Straube and Raus 2014). *Campanula* L. is the largest genus in Campanulaceae, consisting of 350–500 species distributed mainly in the Northern Hemisphere (Federov 1957). Among the *Campanula* species, *Campanula zangezura*, discussed in this study, is very valuable as a horticultural plant owing to its attractive flowers. Unfortunately, genome studies of this species have yet to be carried out. Moreover, Campanulaceae are known to have a highly rearranged chloroplast genome (Cosner et al. 1997, 2004; Haberle et al. 2009; Cheon et al. 2017). Therefore, the structure and sequences of the plastid genome are useful data for clarifying their phylogeny and evolutionary tendencies. However, the cp genome data remain insufficient to clarify the phylogenetic relationships of Campanulaceae.

In this study, we report the complete chloroplast genome sequence of *C. zangezura* to provide genetic information that can be used in various studies in the future. The plant materials for this study were used after seeding and cultivation in the greenhouse of Kangwon National University. A voucher specimen was deposited into the Kangwon National University Herbarium (KWNU), with voucher number KWNU89889. Total DNA was extracted using a DNeasy Plant Mini Kit (Qiagen Inc., Valencia, CA, USA). Genomic DNA was used for sequencing with the Illumina Miseq (Illumina Inc., San Diego, CA, USA) platform. *C. zangezura* was sequenced to produce 6,736,470 raw reads and 301 bp were obtained from them. The plastid genome assembly was performed by the *de novo* assembly protocol (Cho et al. 2015) via the Phyzen bioinformatics pipeline (http://phyzen.com). Annotation of the chloroplast genome was based on an online available program, DOGMA (Wyman et al. 2004), coupled with manual corrections for start and stop codons. We also compared each gene to the published complete chloroplast genome sequence of Campanulaceae for correct gene annotation. The tRNAs were confirmed using tRNAscan-SE (Schattner et al. 2005). A circular plastid genome map was drawn using the OGDRAW program (Greiner et al. 2019).

The complete chloroplast genome of *C. zangezura* (GenBank accession no. MN756013) is a circular DNA molecule 166,330 bp in length with 38.9% G + C content, composed of a large single-copy (LSC) region of 100,034 bp, a small single-copy (SSC) region of 7781 bp, and two inverted repeat (IR) regions of 29,258 bp. The whole cp genome of *C. zangezura* was 3011 bp and 3221 bp shorter than those of reported two *Campanula* species, *C. punctata* and *C. takesimana*, respectively. The plastid genome contains a total of 113 unique genes constituting 79 protein-coding genes, 30 transfer RNA (tRNA) genes, and 4 ribosomal RNA (rRNA) genes. Among the 113 genes, four genes (*rpl23*, *ycf15*, *infA* and *clpP*) were identified as pseudogenes, and 17 genes (*trnA-UGC*, *trnG-UCC*, *trnI-GAU*, *trnK-UUU*, *trnL-UAA*, *trnV-UAC*, *atpF*, *clpP*, *ndhA*, *ndhB*, *petB*, *petD*, *rpl2*, *rpl16*, *rpoC1*, *rps12*, and *ycf3*) contain one or two introns.

To investigate the phylogenetic relationships between *C. zangezura* and related taxa, we conducted a phylogenetic analysis. The cpDNA sequence except for the IGS (intergenic spacer region) due to many rearrangements was used to determine the relationships among the Campanuloid species. A total of 76 protein-coding gene sequences from nine Campanuloid species and one outgroup (*Wahlenbergia marginata*) were aligned using MAFFT (Katoh et al. 2002). We then a conducted maximum likelihood (ML) analysis using RAxML v.7.4.2 with 1000 bootstrap replicates and the GTR + I model (Stamatakis 2006). The ML tree (Figure 1) showed that Campanuloid species formed two clades, the *Campanula* s. str. clade and the *Rapunculus* clade. In addition, *C. zangezura* formed a sister to the *C. punctata* and *C. takesimana* clade in the *Campanula* s. str. clade with a high support value (BP = 100).

**Figure 1.**
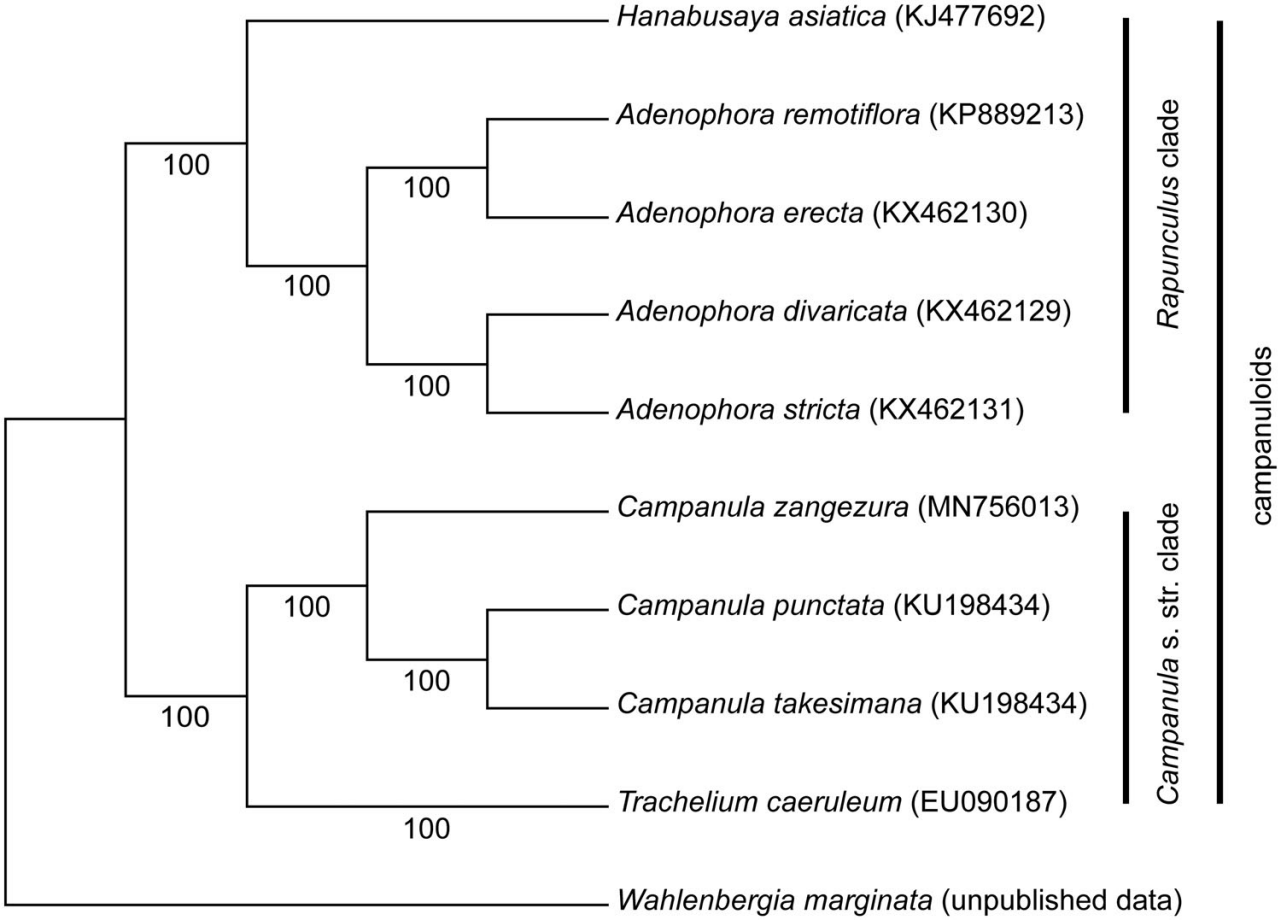
The ML tree based on 76 protein-coding genes from eight Campanuloid species and an outgroup.
